# Solvation‐Mediated Shift From Solvent‐ to Anion‐Derived Solid Electrolyte Interphases for Stable Calcium Metal Anodes

**DOI:** 10.1002/advs.74912

**Published:** 2026-03-20

**Authors:** Yunyun Gao, Jinlei Zhang, Shu Yang, Mengyuan Zhou, Ruimin Li, Zhenghui Pan, Zheng‐Long Xu, Xiaowei Yang

**Affiliations:** ^1^ School of Materials Science and Engineering Tongji University Shanghai China; ^2^ School of Chemistry and Chemical Engineering Shanghai Jiao Tong University Shanghai China; ^3^ Research Institute for Advanced Manufacturing Department of Industrial and Systems Engineering The Hong Kong Polytechnic University Hong Kong China; ^4^ Shenzhen Research Institute The Hong Kong Polytechnic University Shenzhen China

**Keywords:** calcium metal batteries, Ca^2+^ solvation structures, solid‐electrolyte interphase, solvation regulating strategy

## Abstract

Calcium metal batteries represent a promising next‐generation energy storage device due to the high abundance and high capacity of Ca, but remain almost irreversible in ether‐based electrolytes. By elucidating the Ca^2^^+^ solvation structures, we uncover that these detrimental passivation components originate from thermodynamically unstable Ca^2^^+^–DME coordination, which promotes the formation of highly reactive solvent‐separated ion pairs (SSIPs). This understanding motivates a co‐solvent design strategy that reshapes the coordination environment through selective solvent capture using ionic liquids. Guided by theoretical screening, 1‐ethyl‐3‐methylimidazolium bis(trifluoromethylsulfonyl)imide (EMIMTFSI) is identified as the most effective additive. In the optimized electrolyte, strong interactions between EMIM^+^ and DME reduce the population of unstable SSIPs, while TFSI^−^ anions are preferentially incorporated into Ca^2^^+^ solvation shells, yielding a contact‐ion‐pair (CIP)‐rich configuration. This fundamentally switches the electrolyte decomposition pathway from solvent‐derived organics to an inorganic‐rich SEI from TFSI^−^ fragments, thus resulting in a denser and ion‐permeable solid‐electrolyte interphase on Ca metal surface. Consequently, Ca||Ca symmetric cells exhibit stable cycling for 160 h with low overpotentials, whereas the base electrolyte fails after the initial cycle. Further improvement is achieved by replacing TFSI^−^ with more compatible OTf^−^ anions, extending stable cycling to 180 h at an overpotential of 0.3 V.

## Graphical abstract

An ion‐dipole interaction based strategy is proposed to capture thermodynamically unstable solvents and restructure the coordination environment of calcium ions by introducing ionic liquid co‐solvents, thus suppressing excessive solvent decomposition. Therefore, a smooth and dense solid‐electrolyte interphase dominated by inorganic components is formed and better calcium plating/stripping performance is achieved.

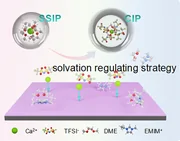

## 1 Introduction

Rechargeable calcium (Ca) metal batteries (CMBs) have emerged as compelling candidates for the next‐generation energy storage beyond‐lithium‐ion batteries owing to the intrinsic merits of Ca metal anode, including enhanced safety, high natural abundance, high volumetric capacity (2073 mA h cm^−3^), and low reduction potential (−2.87 V vs. standard hydrogen electrode) [1, 2, 3, 4, 5, 6, 7, 8, 9, 10, 11, 12, 13]. To take privilege of these advantages in practical CMBs, the establishment of suitable electrolyte that is capable of supporting highly reversible Ca^2+^ plating/stripping [14, 15]. Conventional electrolytes containing simple salts in ether or ester solvents offer attractive thermal stability and chemically benign, non‐nucleophilic environment [16, 17]. However, these formulations suffer from serious Ca‐surface passivation arising from the thermodynamically unstable solvated species. The resulting electron‐conductive and ion‐blocking interphases largely impedes Ca^0^/Ca^2+^ redox, leading to increasing overpotentials and rapid cell failure [18, 19, 20, 21]. Although recent progress has demonstrated Ca^2+^‐conductive and electron‐insulative interphase in novel electrolyte systems [22, 23, 24], the fundamental mechanisms governing passivation evolution and the strategies to mitigate its chemical origins remain poorly understood.

The double charge of divalent Ca^2+^, while is essential for achieving high theoretical energy density, inevitably leads to the strong electrostatic interaction between Ca^2+^ and solvents and anions. These interactions result in complex solvation environment in bulk electrolytes and imposes sluggish desolvation dynamics at the Ca metal anode surface, both of which contribute to high Ca^2+^ plating/stripping overpotentials [25, 26]. Moreover, during desolvation, the coordinating solvent inevitably undergo reductive decomposition and produce organic‐rich components in the passivation layer [27] that further inhibit Ca^2+^ transport and accelerate the failure of Ca metal anodes [28]. Well‐established perception suggests that the coordinated or bound solvents (e.g., DME molecule) in the solvation structures are more susceptible to decomposition than free solvent due to the strong cation chelation and enhanced nucleophility [21, 29], especially for the multivalent‐ion battery systems. Despite this speculation, detailed insights into how Ca^2+^ coordination effects on the structural evolution, stability, and thermodynamic properties of the solvation sheath remain limited. Since electrolyte composition (i.e., solvation structure) and its behavior on the electrode may be the primary factors influencing electrode performance [30], this knowledge gap is essential for developing effective strategies in suppressing solvent breakdown and enhancing the reversibility of Ca^2+^ plating/stripping.

In this work, using the widely affordable Ca(TFSI)_2_/1,2‐dimethoxyethane (DME) electrolyte, our theoretical calculations reveal that the strong coordination interaction between Ca^2+^ and DME oxygen atoms promotes the formation of solvent‐separate ion pairs (SSIP, i.e., [Ca(DME)_4_]^2+^) (Figure S1). We then find that these tightly bound DME molecules are significantly more prone to reduce than the uncoordinated solvent, thus producing an unfavorable organic‐rich SEI that severely limits the interfacial stability during Ca^2+^ plating/stripping. Building on this mechanistic insight, we then introduce ionic liquid (IL, exemplified by EMIMTFSI) co‐solvents to modulate the Ca^2+^ coordination environment. The IL components (EMIM^+^) weakens the Ca^2+^‐DME interactions and suppresses SSIP formation, while TFSI^−^ anions competitively coordinate with Ca^2+^ by displacing the DME from the primary solvation sheath. This solvation restructuring steers interfacial decomposition pathways toward forming an inorganic‐rich SEI with high electronic insulation and ionic conduction properties. Consequently, the optimized electrolyte achieves a markedly reduced Ca^2+^ plating/stripping overpotentials to 0.6 V and a prolonged life span above 160 h compared with the overpotential >5 V and life span ≤8 h in the base electrolyte. In addition, the generality of this approach is further demonstrated by using OTf^−^‐based electrolyte, where similar solvation regulation and anion‐driven inorganic SEI formation effectively suppress solvent decomposition and enable more reversible Ca metal anodes.

## 2 Results and Discussion

### 2.1 Revealing the Intrinsic Correlation Between the Original Passivation Layer and Thermodynamically Unstable Bound Solvents

**FIGURE 1:**
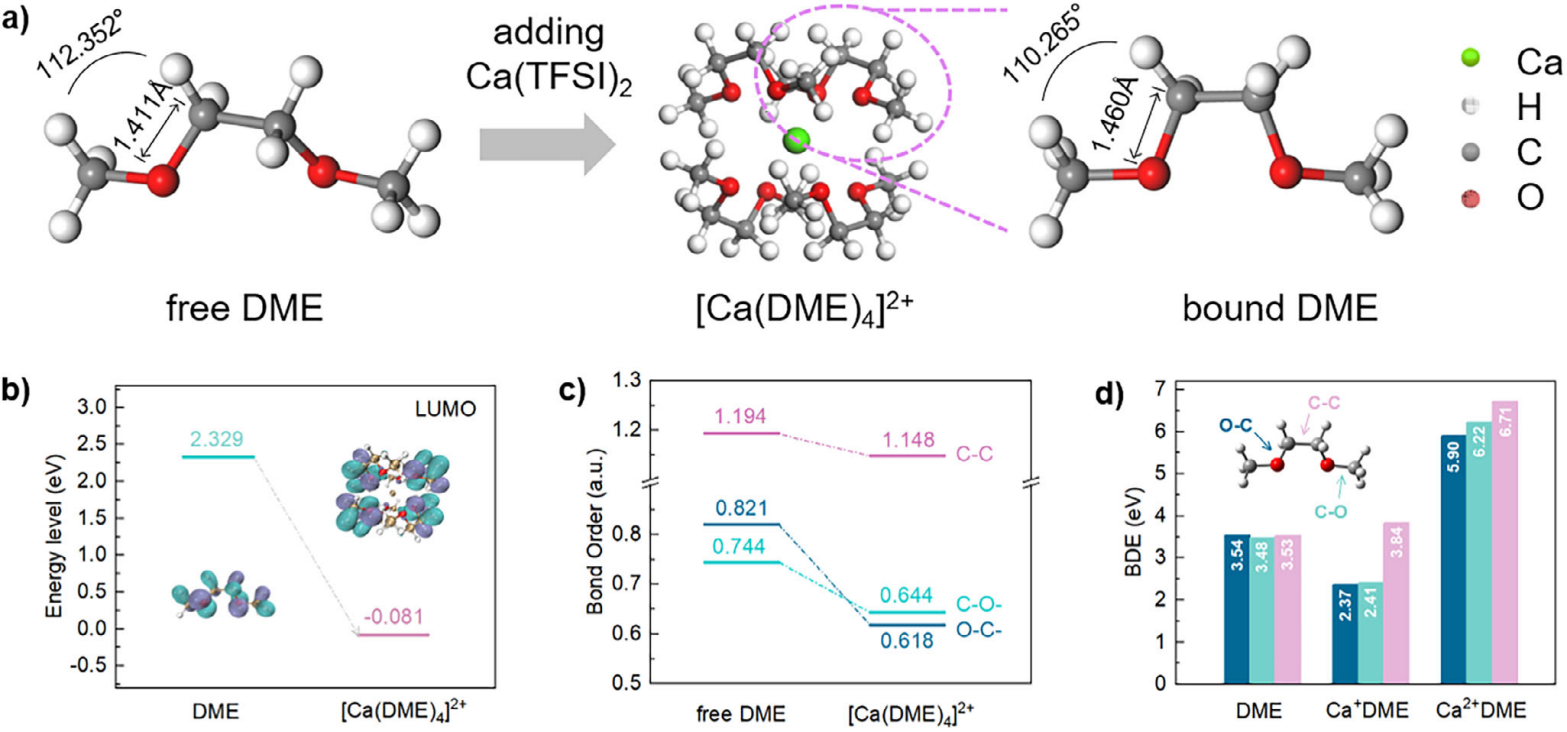
(a) Scheme of the CTFSI‐DME solvation structure and the corresponding characteristics of the free DME and bound DME. (b) The LUMO of free DME and [Ca(DME)_4_]^2+^. (c) The bond orders of different bonds in DME given by DFT calculations. (d) BDE of DME in different chemical environments corresponding to well‐solvated, Ca^+^ ion‐paired and Ca^2+^ ion‐paired configurations.

To explore the intrinsic correlation between the passivation layer on the Ca metal surface and Ca^2+^‐DME coordination, systematic density functional theory (DFT) calculations were first conducted. This investigation focused on the stabilization of DME in both free and bound states, while particularly examining cation‐mediated effects on DME decomposition. Marked by a large ionic radius and high charge, the distinctive characteristics of Ca^2+^ engender strong coordination interactions between Ca^2+^ and oxygen atoms in DME [31, 32, 33]. These interactions lead to the predominant formation of SSIP complexes (i.e., [Ca(DME)_4_]^2+^) in the solvation structure of the 0.1 M Ca(TFSI)_2_/DME electrolyte (marked as CTFSI‐DME). The proportion of different solvation structures in CTFSI‐DME can be found in Figure S1. The coordination interaction induces structural distortions in DME molecules manifested by elongation of C─O bonds and contraction of the C─O─C bond angle. In detail, the length of the C─O bond changes from 1.411 to 1.460 Å, and the angle of the C─O─C bond decreases from 112.352° to 110.265° (Figure 1a). Table S1 summarizes the charge distributions of free and bound DME by Natural Bond Orbital (NBO) analysis. The data reveal that the Ca^2+^‐DME coordination interaction significantly impacts the electronic structure of the solvent molecules, leading to the structural deformation of DME. The electron transfer from coordinated DME to Ca^2+^ with 0.24 e^−^. These structural and electronic modifications collectively destabilize the DME configuration. These changes in molecular structure and charge make it convert into an unstable state. As a result, the lowest unoccupied molecular orbital (LUMO) energy level of DME decreases from 2.329 eV (free state) to −0.081 eV (coordinated state) (Figure 1b), which again demonstrates that the coordinated DME is easier to reduce to organic‐rich components. Figure 1c shows the bond orders of different states of DME. The calculated C─O, O─C, and C─C bond orders all decreased, indicating that DME tends toward an unstable state after binding. Among the three types of bonds in DME, the most pronounced attenuation occurred in the O─C bond order, from 0.821 (free state) to 0.618 (coordinated state), again suggesting that coordinated DME tends to preferentially break the O─C bond during decomposition.

To go deep into the cation effect on the stability of coordinated DME, we further calculated the bond dissociation energy (BDE) for the ion pair Ca‐DME under varying reduction states. As shown in Figure 1d, the bond dissociation energies of the three bonds in coordinated DME are all high when combined with Ca^2+^. However, after the ion pair obtains an electron during the reduction process, the BDE of the C─O and O─C bonds decreases significantly compared to the corresponding bonds in the free state and the ion pair Ca^2+^‐DME. Among them, the dissociation energy of the O─C bond decreases the most, from 3.54 eV to 2.37 eV, proving that the stability of the DME bonds is influenced by the cation during the reduction process. This metastable configuration facilitates preferential O─C bond cleavage, ultimately driving the decomposition of DME from the O─C bond. DME and Ca^2+^ are co‐reduced and decomposed to form an inorganic‐poor passivation layer, which makes the ratio of inorganic and organic components unbalanced. Excessive organic carbon components on the surface lead to poor electronic insulation properties, resulting in irreversible side reactions that rapidly hinder the Ca^2+^ plating/stripping. Thus, reducing coordinated DME in the Ca^2+^ solvation sheath could stabilize electrolyte configurations, optimize passivation composition, and ultimately promote reversible Ca^2+^ plating/stripping.

### 2.2 Solvation Regulating Strategy to Restructure the Ca^2+^ Coordination Environment

**FIGURE 2:**
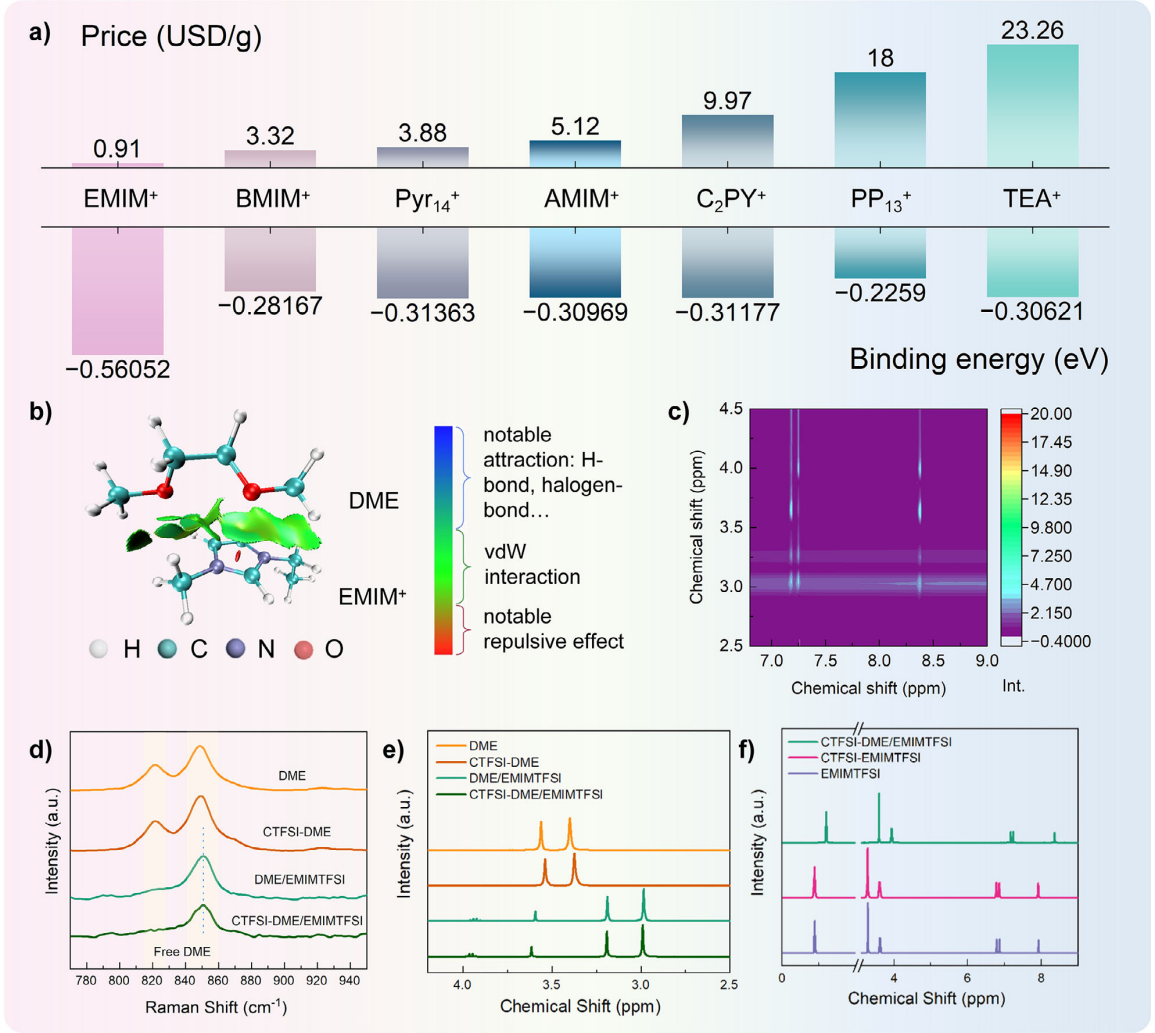
(a) The characteristic comparison of different ILs (XTFSI, X = EMIM^+^, BMIM^+^, Pyr_14_^+^, AMIM^+^, C_2_PY^+^, PP_13_^+^, TEA^+^) based on their prices and binding energy values with DME solvent. (b) IRI isosurfaces of EMIM^+^‐DME. The blue IRI isosurface indicates the presence of strong hydrogen bonding; the green color indicates the presence of robust van der Waals interaction, and the red color indicates obvious intermolecular steric hindrance. (c) Enlarge the NOESY spectrum in the range of 6.8–9.0 ppm on the horizontal axis and 2.5–4.5 ppm on the vertical axis. (d) Raman spectra of the two electrolytes. (e) Single‐pulse ^1^H‐NMR spectra of DME. (f) Single‐pulse ^1^H‐NMR spectra of EMIM^+^.

To restructure the Ca^2+^ solvation structure and realize an inorganic‐rich SEI on the Ca anode surface, a strategy of ion‐dipole interaction between cation and solvent was proposed [34, 35, 36, 37, 38]. It should be noted that new problems could happen if additional metal cation salts are introduced, such as limited salt solubility and easier coordination of metal cations with anions in the electrolyte, which make it unable to play an effective role. Thus, ILs with superior solubility and high ionic conductivity are ideal candidates [39, 40, 41], and we initially screened a series of representative IL cations for investigation by DFT calculations. To eliminate interference from additional anion‐derived SEI heterogeneity, all IL systems employed anions identical to those in the calcium salt. First, Figure 2a presents and compares the cost per gram and binding energies with DME for several commercially available ILs based on TFSI^−^ anions, and the molecular models are shown in Figure S2. It can be found that the EMIMTFSI demonstrates superior cost‐effectiveness (lowest price per gram) and the corresponding EMIM^+^ cation exhibits the strongest binding energy with DME among these candidates. Mixed electrolyte of DME and EMIMTFSI has higher ionic conductivity (Figure S3). Thus, the EMIMTFSI IL was selected as the targeted co‐solvent to be added to the base electrolyte. The Interaction Region Indicator (IRI) is a method that can visually characterize the interaction between atoms in chemical systems. As shown in Figure 2b, a strong van der Waals interaction between EMIM^+^ cation and DME solvent depicted by a large area of green IRI isosurface can be observed. In addition, to further analyze the interaction, the charge distribution of the two molecules was also quantified through the electrostatic potential (ESP) calculation (Figure S4). Because DME is more electron‐rich at O while EMIM^+^ is more electron‐deficient at N, the ion‐dipole interaction between EMIM^+^ and DME is formed through the positively chanrged N and electronegative O.

To better verify the effect of EMIMTFSI on restructuring the Ca^2+^ solvent coordination via ion‐dipole interactions, we perform a series of experimental and theoretical investigations. Raman spectra in Figure 2d show that the peaks observed at 821.3 and 848.3 cm^−1^ are attributed to free DME. After the addition of EMIMTFSI, one of the free DME peaks decreases, proving that the addition of EMIMTFSI resulted in a change in the Ca^2+^ solvation structure in the CTFSI‐DME/EMIMTFSI electrolyte system. Moreover, the peak of coordinated DME (869.5 cm^−1^) shifts to a lower wavenumber and the intensity increases, implying that Ca^2+^‐DME interactions become weaker and DME has coordination with EMIM^+^ via ion‐dipole interaction. Figure 2e,f provides single‐pulse ^1^H‐NMR spectra of DME, DME/EMIMTFSI, EMIMTFSI and two different electrolytes. Based on the combined analysis of Table S2 for the ^1^H NMR spectra of DME and EMIM^+^, the addition of Ca(TFSI)_2_ exerts negligible effects on the ‐CH, ‐CH_2_, and ‐CH_3_ groups in both components. However, the interactions between DME and EMIMTFSI induce substantial shifts in the resonance peaks of DME's ‐CH_2_/‐CH_3_ groups (shifted from 3.56 and 3.40 ppm to 3.19 and 2.99 ppm, respectively) and EMIM's ‐CH/CH_3_ groups (shifted from7.93 and 3.29 ppm to 8.39 and 3.62 ppm), indicating pronounced ion‐dipole interactions between the two species. After the addition of EMIMTFSI, the resonance frequency of DME was upfield‐shifted from the origin peak, which indicates that the ion‐dipole interaction between DME and EMIM^+^ leads to a decrease in the charge density of H in DME. Furthermore, we conducted ^1^H‐^1^H NOESY spectroscopy on the modified electrolyte to more directly visualize the ion‐dipole interactions between EMIM^+^ and DME [42]. As illustrated in Figure S5a, the two singlets of DME exhibit pronounced upfield shifts, while all peaks of EMIMTFSI display significant downfield shifts. This indicates intermolecular interactions between DME and EMIMTFSI that induce substantial chemical shift changes in their respective ^1^H NMR spectra. These interactions were further corroborated by NOESY experiments (Figure S5b), where cross‐peaks manifest for protons within <5 Å spatial proximity. Magnification of the region (6.8–9.0 ppm horizontal, 2.5–4.5 ppm vertical) in Figure 2c and Figure S5c reveal correlations between methyl/methylene protons of DME and ring protons of the imidazolium moiety in EMIMTFSI, confirming their spatial proximity (<5 Å). Upon intensity adjustment, correlations are observed between DME methyl/methylene protons and ethyl group protons of EMIM^+^; DME methyl protons and methyl group protons of EMIM^+^ (designated region, Figure S5d). Integrated analysis of 1D ^1^H NMR chemical shift perturbations and NOESY data thus confirms spatial adjacency between DME and EMIM^+^, satisfying the distance criterion for ion‐dipole interactions. This demonstrates detectable ion‐dipole interactions between EMIM^+^ and DME.

### 2.3 Analysis of Ca^2+^ Solvation Structures in Different Electrolytes

To better confirm the restructured solvation sheath of Ca^2+^ in different electrolytes, we first performed X‐ray absorption fine structure (XAFS) analysis, which has been demonstrated as a powerful and solid technology to investigate the local structures of materials. Figure S6a displays the Ca K‐edge X‐ray absorption near‐edge structure (XANES) spectra of CaO and the electrolytes mentioned above. The near‐edge absorption energy of CTFSI‐DME/EMIMTFSI is lower than that of CTFSI‐DME, indicating a decrease in charge density around Ca^2+^ due to changes in the solvation structure, which is more conducive to the desolvation of Ca^2+^. The Fourier transform spectra of the Ca K‐edge in R space (FT‐EXAFS) for two electrolytes were fitted to provide more detailed confirmation of the coordination structure (Figure S6b). As shown in Table S3, the radius distance of Ca‐O(DME) is 2.31 Å. The addition of EMIMTFSI results in less DME coordination around Ca^2+^. The coordination number of Ca and oxygen in DME decreased, confirming the changes in Ca^2+^ solvation structure, which can be attributed to the reduced content of SSIP in the CTFSI‐DME/EMIMTFSI electrolyte. In addition, Figure S6c provides the wavelet transformed (WT) EXAFS to further prove the change in solvation structure around Ca^2+^. In the CTFSI‐DME electrolyte, the red area where the peaks of R space and K space coincide is located at higher K and R values. In contrast, in the CTFSI‐DME/EMIMTFSI electrolyte, these areas shift toward lower K and R values, indicating an increase in the amount of Ca‐O (TFSI).

**FIGURE 3:**
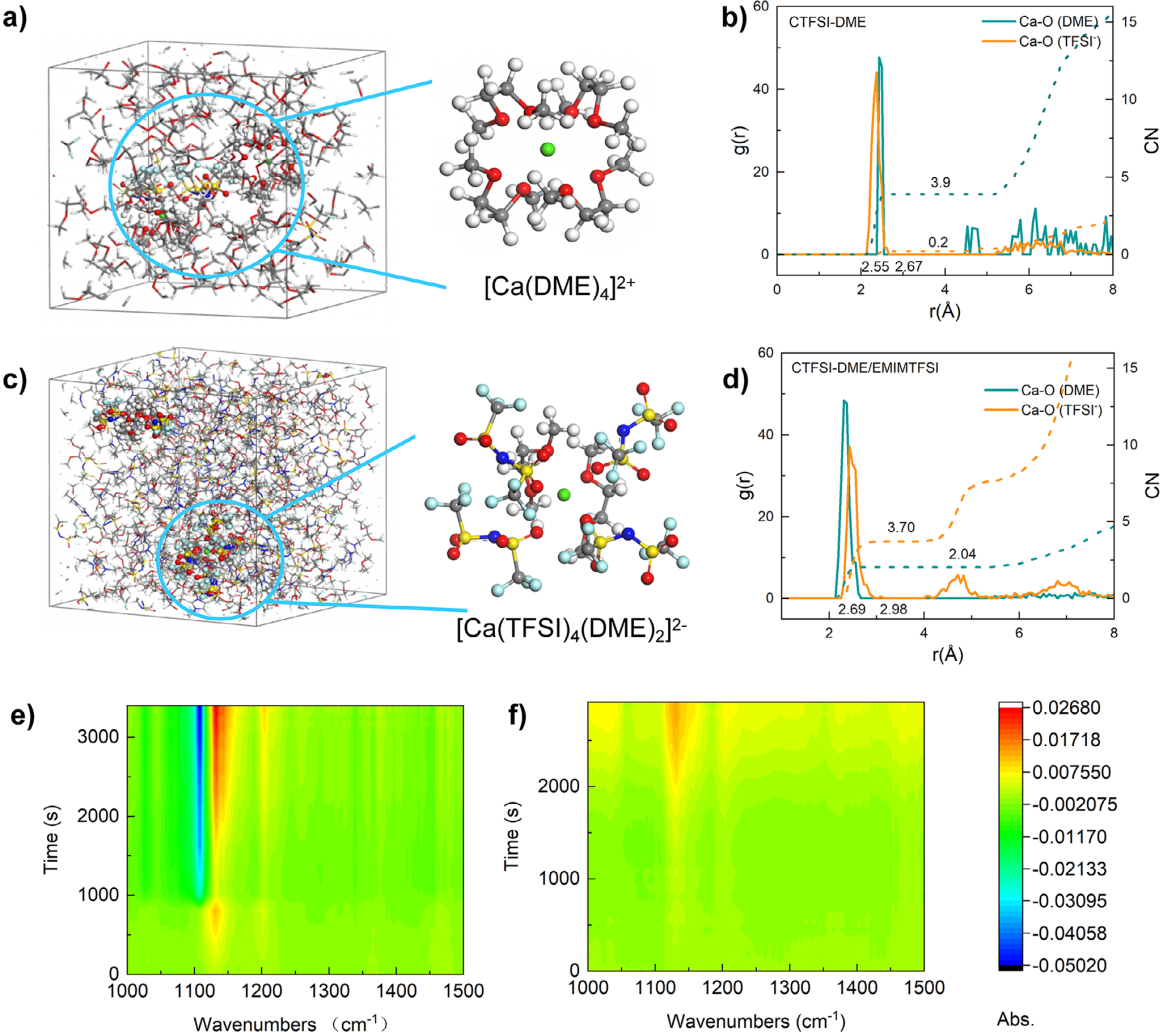
MD snapshots and typical solvation structures of (a) CTFSI‐DME and (c) CTFSI‐DME/EMIMTFSI. The radical distribution function g(r) analyses of (b) CTFSI‐DME and (d) CTFSI‐DME/EMIMTFSI. In situ FTIR spectra for (e) CTFSI‐DME and (f) CTFSI‐DME/EMIMTFSI.

Next, molecular dynamics (MD) simulations were carried out to provide more detailed information on the solvation structures of CTFSI‐DME and CTFSI‐DME/EMIMTFSI electrolytes. Radial distribution function (RDF) analysis of the CTFSI‐DME electrolyte (Figure 3a) reveals an average DME coordination number of 3.9 for Ca^2+^. The predominant solvation structure is illustrated in Figure 3a, confirming that the first solvation shell of Ca^2+^ in this electrolyte is primarily occupied by DME molecules. In contrast, with the introduction of EMIMTFSI, the proportion of solvation structures changes, such as the proportion of SSIP (i.e., [Ca(DME)_4_]^2+^) and CIP (i.e., [Ca^2+^(TFSI^−^)_4_(DME)_2_]^2−^) has respectively decreased from 90% to 2% and increased to 46% (Figure 3c; Figure S7). Moreover, compared with CTFSI‐DME electrolyte, the radial distribution functions (RDFs) of CTFSI‐DME/EMIMTFSI electrolyte show that the number of coordinated TFSI^−^ noticeably increased (Figure 3b,d). Most of the coordinated DME in the Ca^2+^ coordination shell is replaced by TFSI^−^ because of the strong ion‐dipole interaction between EMIM^+^ and DME continuing to form anion‐rich solvation structures. The main solvation structure alteration of the two electrolytes is shown in Figure S8. As expected, the bond length and bond angle of coordinated DME in the solvation sheath tend to be more stable compared to them in CTFSI‐DME electrolyte (Figure S9). Furthermore, Figure S10a‐c reveal the coordination environments of Ca and EMIM^+^ around O(DME) in different electrolytes. This reduction in the average Ca coordination number around DME in the CTFSI‐DME/EMIMTFSI electrolyte is attributed to the ion‐dipole interactions between N(EMIM^+^) and O(DME). These findings further confirm the altered number of coordinated DME molecules in the solvation structure. In addition, due to the high charge of divalent calcium, the non‐polarized force field may lead to overestimation of ion attraction. We conducted additional MD simulations with charge scaling applied to ionic species to investigate the influence of polarizability on coordination behavior and solvation structure composition (Figure S11). Although relative changes occurred in the coordination numbers of Ca^2+^‐O(DME) and Ca^2+^‐O(TFSI^−^) upon accounting for polarizability, the overall coordination trends remained consistent. Within the CTFSI‐DME/EMIMTFSI electrolyte, the prevalence of the [Ca(DME)_4_]^2+^ solvation structure decreased from 98% to 28%. The remaining 72% involved anion participation, effectively reducing the involvement of solvents in the solvation structure.

To further provide a more intuitive demonstration of DME decomposition in the CTFSI‐DME system and the effective suppression of this decomposition in the modified electrolyte by the solvation regulating strategy, we also conducted in situ FTIR measurements during the electrochemical process. Figure S12 displays the electrochemical process during the FTIR test. We scanned from the open‐circuit potential (OCP) to −1 V at a rate of 1 mV s^−1^ to observe electrolyte decomposition. In the base electrolyte, continuous decomposition is observed without any metal stripping current response. In contrast, the modified electrolyte exhibits significantly suppressed decomposition in the early stage and a distinct calcium plating peak near −0.6 V. The FTIR results align with the linear sweep voltammetry (LSV) data. Figure 3e,f shows the temporal evolution of the peak around 1110 cm^−1^, corresponding to the C‐O‐C stretching vibration of DME. In the base electrolyte, the gradual decrease in peak intensity indicates bond cleavage and decomposition of DME at the interface, consistent with the simulated bond‐breaking analysis. In the CTFSI‐DME/EMIMTFSI electrolyte, ion‐dipole interactions between EMIM^+^ and DME effectively suppressed solvent decomposition. Additionally, the peak near 1130 cm^−1^, assigned to the O = S = O symmetric stretching vibration of TFSI^−^, shows a slower intensity increase in the modified electrolyte. This suggests that TFSI^−^ anions in the solvation structure decompose more readily than DME at the interface, facilitating the formation of an SEI with higher inorganic content. Additionally, we conducted an electrolyte exchange experiment [43, 44, 45] (Figure S13). The results indicate that Ca^2+^ desolvation plays a more dominant role in governing Ca deposition/stripping overpotential and cycling stability than the SEI film. Furthermore, the EMIMTFSI‐induced Ca^2+^ solvation restructuring is critical.

Combining the results of characterization and calculation above, it can be concluded that a large amount of DME and EMIM^+^ attract each other in the bulk phase of CTFSI‐DME/EMIMTFSI electrolyte. The introduction of the IL also induces a rise in TFSI^−^ anion density, thereby amplifying the coordination barrier between DME and Ca^2+^. Therefore, the number of DME entering the Ca^2+^ solvation sheath decreases significantly, reducing the presence of thermodynamically unstable coordinated DME. Concurrently, TFSI^−^ anions increase to form a CIP‐rich electrolyte [46], constructing a smooth and dense inorganic‐rich SEI, which will discussed in detail in the following section.

### 2.4 Characterizing and Comparing the Interface Morphologies and Components of Ca Anode Formed in Different Electrolytes

**FIGURE 4:**
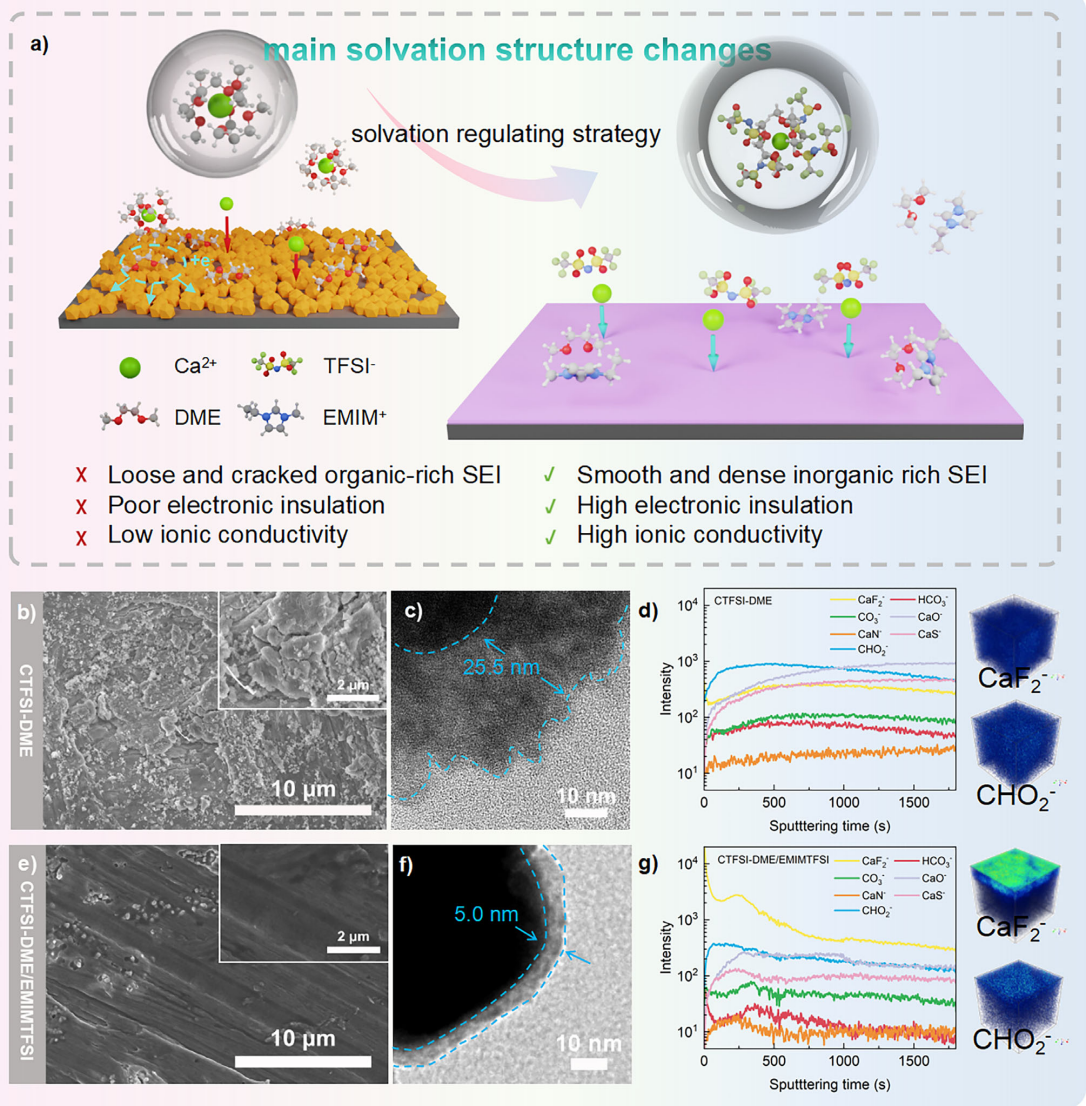
(a) Schematic illustration of solvation regulating strategy restructuring the Ca^2+^ solvent coordination to the formation and evolution of SEIs in CTFSI‐DME and CTFSI‐DME/EMIMTFSI electrolytes. SEM images of the fifth cycled and 15th cycled Ca metal surface (b) in CTFSI‐DME and (e) in CTFSI‐DME/EMIMTFSI electrolytes, respectively. Typical TEM images of the surface interphase from the Ca deposit in (c) CTFSI‐DME and (f) CTFSI‐DME/EMIMTFSI electrolytes. Intensity sputter profiles of SEI species derived from (d) CTFSI‐DME and (g) CTFSI‐DME/EMIMTFSI electrolytes measured by TOF−SIMS and corresponding 3D reconstruction distribution mappings on the Ca metal anode surface after 10 cycles.

Figure 4a depicts the schematic illustration of solvation regulating strategy restructuring the Ca^2+^ solvent coordination to the formation and evolution of SEIs in CTFSI‐DME and CTFSI‐DME/EMIMTFSI electrolytes. In the designed electrolyte, the high‐coordination‐energy N atoms in the EMIM^+^ group can interact strongly with DME's O atoms via ion‐dipole interactions, while TFSI^−^ anions are preferentially incorporated into the primary solvation structures, both which restructure the Ca^2+^ main solvation structure from [Ca(DME)_4_]^2+^ to [Ca^2+^(TFSI^−^)_4_(DME)_2_]^2−^, further promoting a smooth and dense inorganic‐rich SEI for reversible Ca^2+^ plating/stripping.

The morphologies and components of the Ca surface cycled in the two electrolytes were thus characterized to understand the effect of SEI on the electrochemical performance of the Ca metal anode. Scanning electron microscopy (SEM) was first employed to compare the morphologies of SEI layers formed in CTFSI‐DME cycled to the protection potential (only after 5 cycles) and CTFSI‐DME/EMIMTFSI after 15 cycles. Figure 4b reveals a substantial accumulation of porously loose and cracked organic‐rich by‐products on the Ca surface cycled in the base electrolyte, which resulted from the dramatic decomposition of DME solvents. The poor electron‐shielding capability of these organic compounds will facilitate continuous parasitic side reactions at the interface until the depletion of electrolytes [47, 48]. In contrast, the CTFSI‐DME/EMIMTFSI electrolyte exhibits a smooth and dense surface even after prolonged cycling (Figure 4e), indicating the formed SEI effectively suppresses the occurrence of parasitic reactions. Transmission electron microscopy (TEM) images further compare the thickness of the SEI layers in two electrolytes (Figure 4c,f). In the CTFSI‐DME electrolyte, an uneven and loosely packed interphase with a thickness range from 22.9 to 30.1 nm can be observed. In contrast, the SEI layer formed in the CTFSI‐DME/EMIMTFSI electrolyte shows a continuous and uniform structure (∼5.0 nm), effectively suppressing interfacial side reactions and improving efficient Ca^2+^ platting/stripping. Next, time‐of‐flight secondary ion mass spectrometry (TOF‐SIMS) was utilized to reveal the distribution of SEI chemical compositions. As shown in Figure 4d, the passivation layer of the base electrolyte has a high content of organic components. Specifically, a thick passivation layer containing organic‐inorganic hybrid species is formed, which is well consistent with the TEM results. In contrast, Figure 4g shows that the CTFSI‐DME/EMIMTFSI‐derived SEI dominated by a dense layer of inorganic‐rich species resulted from the reduction of anions with the organic‐phase intensity reduced. Note that fluoride has been demonstrated as an effective electronic insulation component of the SEI layer for reversible metal plating/stripping [49, 50]. The differences in the interphase properties of the two electrolytes are reflected in the 3D spatial distribution of organic components and inorganic species (Figure S14). In the modified electrolyte, elevated CaF_2_ content at the interface forms an inorganic‐rich SEI that effectively impedes the formation of detrimental components (e.g., CaCO_3_) and mitigates further decomposition of organic species (e.g., CHO_2_). The diminished carbon signal attests to an overall decrease in organic constituents on the calcium metal surface.

In addition, physical field simulations were performed based on TOF‐SIMS and SEM characterization results of the SEI to elucidate the relationship between different SEI components and calcium deposition behavior. The calcium plating process was simulated via finite element analysis using COMSOL Multiphysics (Figure S15). The native SEI formed in the base electrolyte, composed predominantly of organic compounds with minor inorganic constituents, exhibits heterogeneity and poor electron‐blocking capability. Consequently, the continuous accumulation of undesirable cracked organic products generated from solvent decomposition disrupts ion flow, leading to preferential deposition at high‐field points. This severely impedes uniform Ca^2+^ deposition on the substrate. In contrast, for the CTFSI‐DME/EMIMTFSI electrolyte, the SEI with high electron‐blocking capability and ionic conductivity regulates Ca^2+^ flux distribution, promoting uniform calcium deposition. Overall, the formation of a smoother and denser inorganic‐rich SEI suppresses parasitic reactions between calcium metal and the electrolyte. This consequently facilitates a more uniform electric field distribution across the calcium substrate.

### 2.5 Electrochemical Performance of Ca Metal Anode in Two Different Electrolytes

**FIGURE 5:**
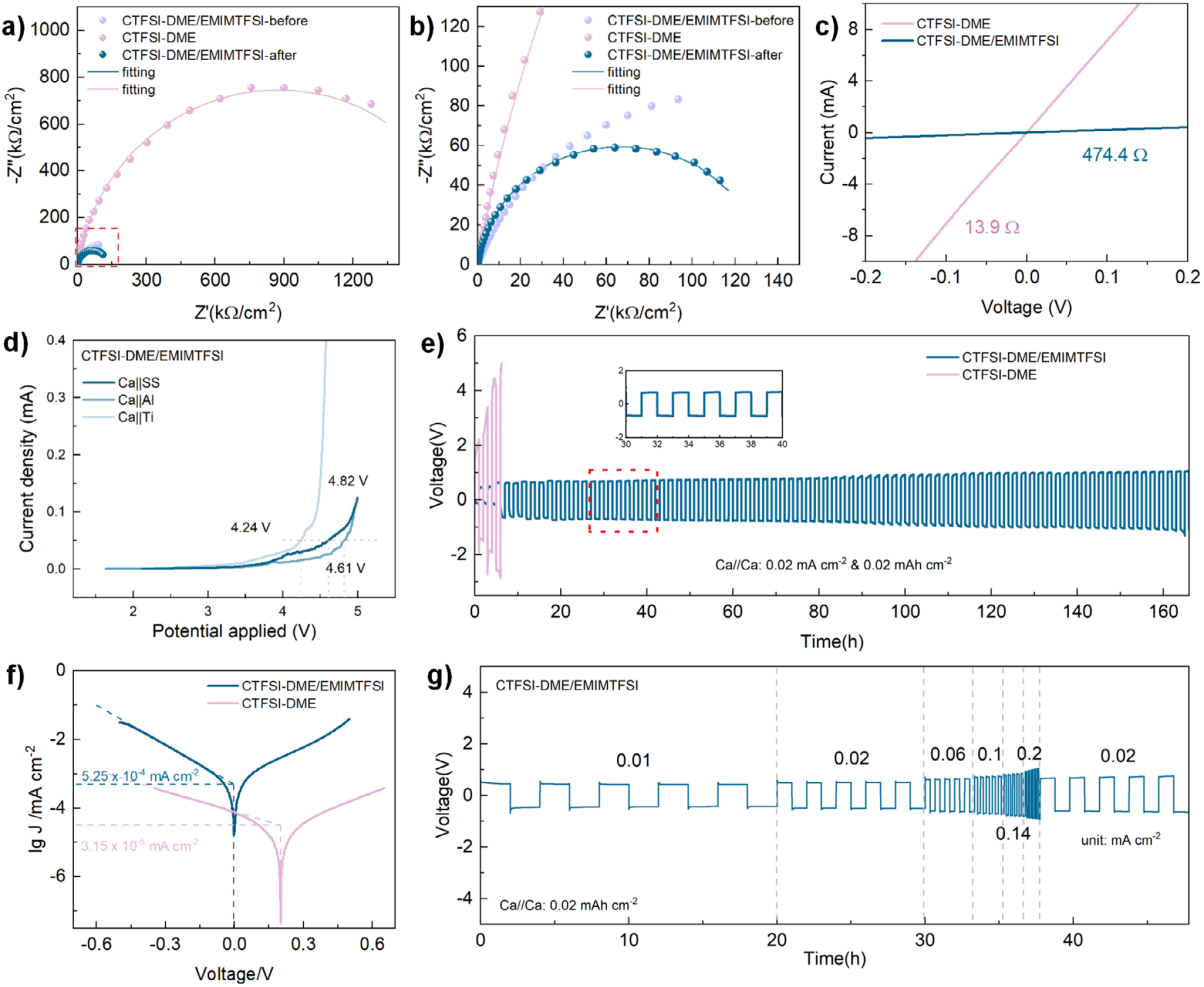
(a) EIS plots of Ca//Ca symmetric cells in two electrolytes before and after cycling. (b) A partially enlarged view of EIS from the red dashed box in the a). (c) LSV curves of Ca electrodes in two electrolytes. (d) LSV of Ca//SS (Al or Ti) asymmetric cells in CTFSI‐DME/EMIMTFSI electrolyte. (e) Galvanostatic cyclic stability of Ca//Ca symmetric cells in two different electrolytes. The inset is the enlarged view of the curve at a specific time region. (f) Tafel plots of Ca//Ca symmetric cells in the two different electrolytes. (g) Rate performance of Ca^2+^ plating/stripping in CTFSI‐DME/EMIMTFSI electrolyte.

We further compare the interface properties of Ca anode formed in different electrolytes by a series of electrochemical performance tests. First, electrochemical impedance spectroscopy (EIS) was carried out and fitted to explore the migration dynamics of Ca^2+^ and the transmission capacity of Ca^2+^ within the interfacial layer in the two electrolytes (Figure 5a,b; Figure S16 and Table S4) [51, 52]. It can be seen that the passivation layer formed in the CTFSI‐DME electrolyte exhibits a very high charge transfer resistance (*R_ct_*). The substantial magnitude (∼ 1750 kΩ) indicates severely sluggish kinetics for Ca^2+^ transport. As interfacial ion translocation precedes charge transfer processes, this kinetic barrier induces pronounced polarization at the anode/electrolyte interface. In contrast, a low *R_ct_* is observed in the initial CTFSI‐DME/EMIMTFSI electrolyte and the value shows an obvious decrease in interfacial resistance (from ∼ 1750 kΩ to ∼ 133 kΩ after 5 cycles) demonstrates the role of SEI layer in promoting Ca^2+^ mobility. This kinetic optimization at the anode/electrolyte interface substantially improves ion transport efficiency. Besides, an ion‐blocking electrode test was used to probe the formed interphase's electronic insulation (Figure 5c). The electronic resistance of the Ca electrode after cycling in the two electrolytes is 13.9 and 474.4 Ω, respectively, which indicates that the SEI formed in the CTFSI‐DME/EMIMTFSI electrolyte has superior electronic insulation, thus effectively suppressing parasitic side reactions of the electrolyte.

To verify the effect of the inorganic‐rich SEI derived from CTFSI‐DME/EMIMTFSI electrolyte on improving reversible Ca^2+^ plating/stripping, the electrochemical performance of Ca anode was systematically performed. First, linear sweep voltammetry (LSV) of the Ca//SS (Al or Ti) asymmetric cell was measured to investigate the electrochemical oxidation stability of the designed electrolyte with different counter electrodes (Figure 5d). All curves exhibit similar oxidation onset potentials across a wide range above 4.4 V, confirming the superior electrochemical stability of the CTFSI‐DME/EMIMTFSI electrolyte. The average electrochemical oxidation potentials for the Ca//SS (Ti or Al) asymmetric cells were 4.495 V, 4.622 V, and 4.815 V, respectively, accompanied by very small standard deviations (Figure S17). This demonstrates the stability of the oxidation window. Then, Figure 5e shows the cyclic stability of Ca//Ca symmetric cells in two different electrolytes under a current density of 0.02 mA cm^−2^ with a capacity of 0.02 mA h cm^−2^, where rapid failure is observed in CTFSI‐DME as evidenced by abruptly degraded cycling profiles within an extremely short timeframe. This result can be attributed to the organic‐rich SEI formed in CTFSI‐DME exhibiting poor electronic conductivity and high interfacial impedance, which leads to sluggish interfacial kinetics and exacerbated polarization. Furthermore, Ca metal platted in CTFSI‐DME electrolytes demonstrates severe stripping difficulties, manifesting as asymmetric overpotential behavior. In contrast, the CTFSI‐DME/EMIMTFSI electrolyte enables symmetric Ca^2+^ platting/stripping behavior with a low voltage hysteresis of 0.6 V without obvious fluctuations, achieving stable cycling for 160 h (80 cycles). Additionally, galvanostatic cyclic stability test conducted at 0.04 mA cm^−2^ exhibit relatively small overpotential (Figure S18). This improved performance demonstrates that the inorganic‐rich SEI constructed by the proposed solvation regulating strategy can largely enhance the cycling stability and reversibility of the Ca metal anode in conventional electrolytes.

To further investigate the Ca^2+^ transport property within the inorganic‐rich SEI, Figure 5f compares the Tafel polarization plots of the two electrolytes. The decreased energy barrier for Ca^2+^ deposition in CTFSI‐DME/EMIMTFSI electrolytes confirms the fast ion transport capability of the inorganics‐rich SEI. Moreover, the CTFSI‐DME/EMIMTFSI electrolyte exhibits a higher exchange current density (*I_0_* = 5.25 × 10^−4^ mA cm^−2^) for Ca^2+^ plating/stripping than that of base one (*I_0_* = 3.15 × 10^−5^ mA cm^−2^), indicating an accelerated Ca^2+^ transport within the optimized SEI layer and fast Ca nucleated process on the Ca anode surface. Finally, the Ca//Ca symmetric cells in the CTFSI‐DME/EMIMTFSI electrolyte also exhibit a superior rate capability with stable platting/stripping overpotential even as the current density increases from 0.01 to 0.2 mA cm^−2^ (Figure 5g), indicating that the designed electrolytes can be employed in different situations with various charging and discharging requirements.

### 2.6 The Generality of the Solvation Regulating Strategy on Restructuring the Coordination Environment for Reversible Ca^2+^ Platting/Stripping

Our proposed solvation regulating strategy for restructuring the Ca^2+^ coordination environment to enable reversible Ca metal anodes suggests potential applicability to other EMIM^+^‐based ILs containing different anions. To provide an initial validation of this concept beyond TFSI^−^ system and to explore the impact of anion functionality on SEI formation, OTf^−^ was chosen specifically because it shares structural similarities with TFSI^−^ but exhibits smaller steric hindrance, while TFSI^−^ decomposition may not fully provide the optimal inorganic components for a highly protective SEI; intermediate organic fragments could even be detrimental [53]. These properties of OTf^−^ are anticipated to lead to more favorable decomposition products, promoting an inorganic‐rich SEI layer that may further enhance Ca plating/stripping kinetics.

**FIGURE 6:**
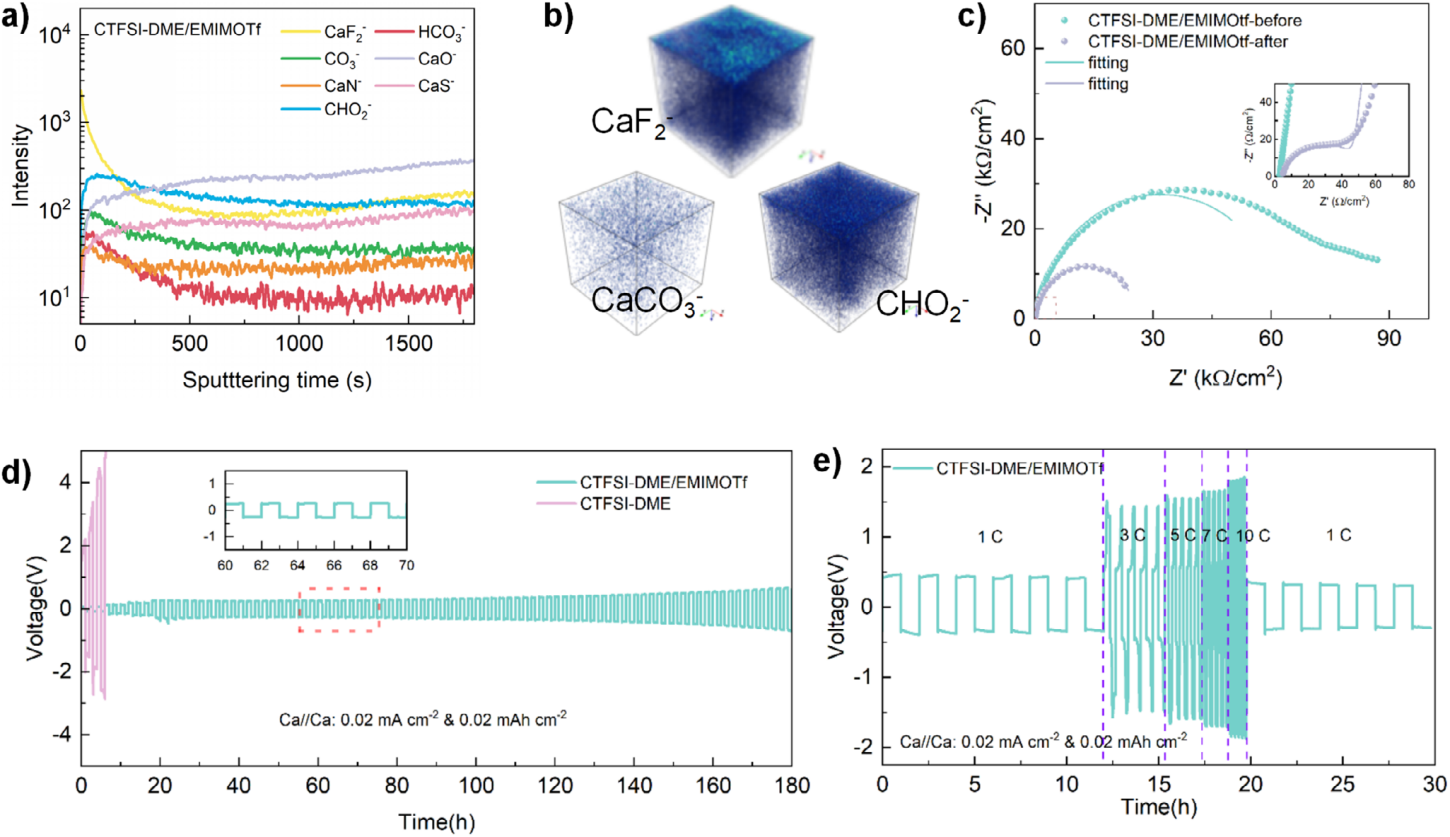
(a) Intensity sputter profiles of SEI species derived from CTFSI‐DME/EMIMOTf electrolyte measured by TOF‐SIMS. (b) Corresponding 3D reconstruction of the distribution mappings on the Ca metal anode surface after 10 cycles in CTFSI‐DME/EMIMOTf. (c) EIS plots of Ca//Ca symmetric cells before and after cycling in CTFSI‐DME/EMIMOTf electrolyte. (d) Galvanostatic cycling stability of Ca//Ca symmetric cell in CTFSI‐DME and CTFSI‐DME/EMIMOTf electrolytes. (e) Rate performance of Ca//Ca symmetric cell in CTFSI‐DME/EMIMOTf electrolyte.

Based on the ion‐dipole interactions between EMIM^+^ and DME, and the stronger attraction between OTf^−^ and Ca^2+^, the number of OTf^−^ anions surrounding Ca^2+^ in the system is significantly higher than that of TFSI^−^ and DME. Furthermore, the outer solvation sheath contains a greater proportion of OTf^−^ (Figure S19). Consequently, as shown in Figure S20, OTf^−^‐enriched solvation structures constitute 82% of the total solvation configurations. These dominant structures include [Ca(DME)_2_(OTf^−^)_4_]^2+^, [Ca(DME)_2_(OTf^−^)_3_]^2+^, and [Ca(DME)(OTf^−^)_5_]^3+^. As expected, TOF‐SIMS depth profiling in Figure 6a,b reveals diminished intensities of various fragments at the interface. Within the formed SEI, inorganic components are dominated by CaF_2_ and CaO. Notably, CaF_2_ exhibits a pronounced intensity decrease with increasing sputtering depth, indicating that OTf^−^ decomposition generates a thin, dense CaF_2_ layer in the SEI. The organic component CHO_2_^−^ (derived from DME) also declines with sputtering depth, whereas organic constituents in the baseline electrolyte persist at elevated levels. The corresponding 3D reconstruction of distribution mappings on the Ca metal anode surface (Figure S14) across the three electrolytes further confirms that CTFSI‐DME/EMIMOTf forms the thinnest SEI, effectively suppressing detrimental side reactions. These compositional changes effectively enhance electronic insulation and yield a smoother, thinner and Ca‐deposition‐homogenizing interphase after cycling (Figure S21). Compared to the CTFSI‐DME electrolyte induced massive accumulation of bulky organic aggregates on anode surfaces, the SEM shows the modified electrolyte with enhanced inorganic constituents promoted the formation of a compact SEI layer, which simultaneously suppresses solvent decomposition pathway, thereby effectively passivating the electrode/electrolyte interface against further parasitic reactions.

The corresponding electrochemical performance in two electrolytes has further been investigated. First, the electrochemical oxidation stability of the CTFSI‐DME/EMIMOTf electrolyte was evaluated by linear sweep voltammetry (LSV) using different electrode materials. To demonstrate the reproducibility of the electrolyte's oxidative stability, the results are presented as data points with error bars. As shown in Figure S22, all electrodes exhibit onset oxidation potentials above 4 V with small standard deviations. Then, the EIS results in Figure 6c indicate a marked decrease in the overall interface resistance (*R_int_*) for the modified electrolyte. Fitting of the EIS data (Figure S23 and Table S5) reveals that the *R_ct_* decreased from 1750 kΩ in the base electrolyte to 63.5 kΩ in the initial CTFSI‐DME/EMIMOTf electrolyte. After five cycles, *R_ct_* further decreased to 26.1 kΩ. Compared to the original Nyquist plot exhibiting a single semicircle, the interphase resistance (*R_SEI_*) post‐SEI formation decreased by five orders of magnitude (to only 42.57 Ω). This demonstrates that the inorganic‐rich SEI formed in the CTFSI‐DME/EMIMOTf electrolyte substantially enhances Ca^2+^ transport across the interface, enabling more rapid interfacial kinetics for Ca^2+^. Furthermore, the exchange current density (I_0_ = 5.02 × 10^−4^ mA cm^−2^) for calcium plating/stripping increased. This may be attributed to the suppression of DME decomposition, which reduced the surface diffusion resistance and optimized the interface (Figure S24). Regarding the cycling stability, the CTFSI‐DME/EMIMOTf electrolyte also enables symmetric plating/stripping behavior with a low overpotential of 0.3 V under 0.02 mA cm^−2^ and 0.02 mA h cm^−2^ (Figure 6d), achieving stable cycling for 180 h (90 cycles), which is even better than the electrolyte that introduces TFSI^−^ anions. The CTFSI‐DME/EMIMOTf electrolyte also demonstrates good cycling performance under 0.04 mA cm^−2^ and 0.04 mA h cm^−2^ (Figure S25). The rate performance is also shown in Figure 6e, where the plating/stripping overpotential does not change significantly when the current density is increased from 0.01 to 0.2 mA cm^−2^ and then returned to the initial state, indicating that the formed SEI has good stability. Collectively, the aforementioned characteristics and electrochemical evaluations demonstrate that the strategy leveraging ion‐dipole interactions of IL cations along with anion‐Ca^2+^ electrostatic attraction to improve solvation structures effectively enhances CMB performance in conventional electrolytes, exhibiting competitive electrochemical properties among existing modification approaches for calcium salts and solvents reported to date (Table S6).

To prove the practical application of the stable Ca anode realized by our designed electrolytes, a 3,4,9,10‐Perylenetetracarboxylic‐diimide (PTCDI)//Ca full cell was assembled (Figure S26). As expected, the PTCDI//Ca full cell delivers a competitive discharge‐specific capacity of 150 mA h g^−1^ and proving the feasibility and reliability of the CTFSI‐DME/EMIMTFSI electrolyte.

## 3 Conclusions

In summary, this work first revealed that the strong Ca^2+^‐DME coordination in conventional electrolytes (e.g., Ca(TFSI)_2_ in DME) thermodynamically triggers DME decomposition and forms a thick and loose organic‐dominated passivation layer with high ion‐blocking resistance and electron‐conduction capability, which results in the failure of the Ca metal anode. Then, a solvation regulating strategy by IL co‐solvent was proposed to regulate the Ca^2+^ solvation sheath; specifically, introducing EMIMTFSI weakens Ca^2+^‐DME binding by EMIM^+^‐DME dipole coupling, reducing the proportion of [Ca(DME)_4_]^2+^ and suppressing solvent degradation. Concurrently, TFSI^−^ anions displace DME from the primary coordination structure to obtain an anion‐rich solvation structure, promoting the formation of a thin, inorganic‐rich SEI, which exhibits high electronic insulation and ionic conduction and effectively blocks parasitic reactions. The optimized SEI chemistry enables stable Ca plating/stripping with a low overpotential and a long‐term lifespan, markedly outperforming the base electrolyte. More significantly, this strategy is further validated its potential applicability beyond the primary anion studied (e.g., substituting TFSI^−^ with OTf^−^), which enhances SEI inorganic content and achieves an even lower overpotential. These findings demonstrate the feasibility of solvation sheath modulation via ion‐dipole interactions and anion selection as a promising strategy for CMBs. Future efforts will focus on validating the broader applicability of this approach. This will involve anion library expansion, the exploration of further IL variants, scalable electrolyte design, and mechanistic studies on interfacial dynamics.

## Conflicts of Interest

The authors declare no conflicts of interest.

## Supporting information




**Supporting File**: advs74912‐sup‐0001‐SuppMat.docx

## Data Availability

The data that supports the findings of this study are available in the supplementary material of this article.
