# Supplementary material for: Solvation‐Mediated Shift From Solvent‐ to Anion‐Derived Solid Electrolyte Interphases for Stable Calcium Metal Anodes
Source: Adv Sci (Weinh). 2026 Mar 20;13(31):e74912. doi: 10.1002/advs.74912 (PMC13587935; doi:10.1002/advs.74912)
Supplement: Supplementary file 1 [file ADVS-13-e74912-s001.docx]

**Supporting Information**

**Solvation-mediated Shift from Solvent- to Anion-derived Solid Electrolyte Interphases for Stable Calcium Metal Anodes**

Yunyun Gao^1+^, Jinlei Zhang^1+^, Shu Yang^1^, Mengyuan Zhou^1^, Ruimin Li^2^, Zhenghui Pan^1*^, Zheng-Long Xu^3,4*^, Xiaowei Yang^2^

^1^School of Materials Science and Engineering, Tongji University, Shanghai 201804, China

^2^School of Chemistry and Chemical Engineering, Shanghai Jiao Tong University, Shanghai 200240, China

^3^ Research Institute for Advanced Manufacturing, Department of Industrial and Systems Engineering, The Hong Kong Polytechnic University, Hung Hom, Hong Kong SAR 999077, China

^4^ Shenzhen Research Institute, The Hong Kong Polytechnic University, Shenzhen, China.

^+^These authors contributed equally to this work

Email addresses: zhenghuipan@tongji.edu.cn; zhenglong.xu@polyu.edu.hk; yangxw@sjtu.edu.cn

**Experimental**

*Electrolyte preparation:* 0.1 M Ca(TFSI)_2_-DME electrolyte: 0.06 g calcium bis(trifluoromethanesulfonyl)imide (Ca(TFSI)_2_, >99.50%, DoDoChem) was dissolved in 1 ml dimethoxyethane (DME, >99.5%, Aladdin). 0.1 M Ca(TFSI)_2_-DME/EMIMTFSI electrolyte: 0.06 g calcium bis(trifluoromethanesulfonyl)imide (Ca(TFSI)_2_, >99.50%, DoDoChem) was dissolved in the cosolvent system of 0.5 ml dimethoxyethane (DME, Aladdin, >99.5%) and 0.5 ml 1-Ethyl-3-Methylimidazolium Bis(Trifluoromethylsulfonyl)Imide (EMIMTFSI, 97%, Aladdin).

*Cell assemble:* All electrochemical performance characterizations were conducted with 2032-type cells. Ca//Ca symmetric cells were assembled with the two Ca electrodes (diameter =16 mm, thickness =1.0 mm), a glass fiber separator (Whatman GF/D, 19 mm in diameter) and 120 μL electrolyte. Ca electrodes recovered from Ca symmetric cells after 20 cycles were sandwiched between two stainless steels (SS) to construct a blocking electrode configuration. PTCDI//Ca coin-type cells were assembled with PTCDI electrode and Ca electrode with 120 μL electrolyte. Note that electrolyte preparation, cathode preparation and cell assembly were performed in a glove box (Vigor, Germany), where the contents of water and oxygen were controlled below 0.1 ppm.

*Electrochemistry:* Ca//Ca symmetric cells were charging/discharging at a current density of 0.02 mA cm^−2^ and a capacity of 0.02 mA h cm^−2^ at room temperature, which was performed by a standard battery tester (Netware, CT4008). The electrochemical impedance spectroscopy (EIS) was carried out on Bio-Logic VMP3, with frequencies from 100 kHz to 10^−1^ Hz with a potential amplitude of 5 mV. The electrochemical oxidation activity of electrolytes was determined by linear sweep voltammetry (LSV) from open circuit potential to 5 V at a scanning rate of 1 mV s^−1^. Stainless steel (SS), aluminium (Al) or titanium (Ti) was used as a working electrode. Full cells were assembled using Ca as anode and 3,4,9,10-perylenetetracarboxylic diimide (PTCDI) as the cathode and tested in CTFSI-DME/EMIMTFSI electrolyte. The commercial PTCDI (98%+, Adamas) was directly used as the active cathode material. The slurry consisting of 30 wt% PTCDI, 50 wt% Super P, and 20 wt% polyvinylidene difluoride was cast onto titanium foil to obtain PTCDI electrodes.

*Characterizations:* For the physicochemical characterizations, the cycled cells were disassembled, and then the recovered electrodes were rinsed three times with DME solvent to remove the residual electrolyte, followed by drying in the glove box overnight. Raman measurements were collected on the Thermo Scientific DXRXI system excited from an Ar laser at 532 nm. The ^1^H-NMR spectra were carried out on a Bruker AV400 spectrometer. The morphology of the Ca anode and corresponding Ca deposits were observed with scanning electron microscopy (SEM, FEI–quanta 250, America). Transmission electron microscopy (TEM) images were performed on ThermoFisher Talos F200X G2. The time-of-flight secondary ion mass spectroscopy (TOF-SIMS) was performed using a TOF-SIMS 5-100 (ION TOF) with a Bi^3+^ gun at 50 keV. For surface analysis, the flood gun is tuned to deliver Bi^3+^ primary ions with a 50×50 μm^2^ raster size. Depth profile experiments are carried out using previous conditions for surface analysis (Bi^3+^, 50×50 μm^2^ raster size) and a 2 kV Cs^+^ gas gun for etching (with a sputtered area of 200×200 μm^2^).

**Computational Methods**

**Finite Element Method Simulations**

The calcium plating process was simulated via finite element analysis using COMSOL Multiphysics. A three-dimensional geometric domain (10 μm cube) was discretized with a free tetrahedral mesh under Extra Fine preset. The simulation framework integrates the Tertiary Current Distribution model to accurately capture the electrochemical deposition process of metallic calcium. The diffusion coefficient for Ca^2+^ ions is set to $\text{D}_{\text{Ca}^{\text{2+}}}$=6.22×10^−12^ m^2^/s, and a current density boundary condition of 5 mA cm^−2^ is applied at the electrode-electrolyte interface. The multiphysics model was formulated by solving the coupled system of material balance equations for ionic species and the electroneutrality constraint within the electrochemical boundary layer.

**Density functional theory (DFT) calculations:**

Density functional theory calculations for geometry optimization were carried out using the Gaussian 16 package^1^. Geometry optimizations and energy calculations were carried out with the wb97xd functional in combination with the 6-31+G(d,p) basis set. The convergence tolerance quality was the default. The solvation effect was considered with the universal solvation model of SMD^2^, with the dielectric constant of DME (ε = 5.5) specified to account for the polar contribution. Based on the optimized structures, the lowest unoccupied molecular orbital (LUMO) energy level was analyzed using the Natural Bond Orbitals (NBO) theory^3^. The electrostatic potential (ESP) of the anions and the interaction region indicator (IRI) were analyzed by Multiwfn software^4^. The binding energy (E_b_) of the solvation structures is calculated as:

*E_b_* = *E_total_* - *E_DME_* - *E_Ca_^2+^*

The adsorption energy (Eads) was calculated by the following equation:

*E_ads_=E_total_ – E_(Ca(111))_ - E_adatoms/molecules_*

Where E_total_ was the total energy of the Ca (111) plane with the adatoms/molecules and E_Ca(111)_ and E_adatoms/molecules_ were the energy of the Ca (111) plane and adatoms/molecules, respectively.

**Molecular Dynamics (MD) simulations:**

Molecular Dynamics simulations conducted in this work were performed using the Large–scale Atomic/Molecular Massively Parallel Simulator (LAMMPS, http://lammps.sandia.gov). Ions and molecules involved in the simulation were optimized by Gaussian16 with a level of wb97xd/6-31+g(d,p) to obtain reasonable structures. The compositions of electrolytes are 2400 DME, 50 Ca(TFSI)_2_; 2400 DME, 1025 EMIMTFSI and 50 Ca(TFSI)_2_; 2400 DME, 1350 EMIMOTf and 50 Ca(TFSI)_2_. The size of the periodic box was 10*10*10 nm^3^. After energy minimization and NPT equilibration, the final density-stabilized simulation boxes measured were 9.3^3^ nm^3^, 9.7^3^ nm^3^, and 9.0^3^ nm^3^ for the three systems, respectively. OPLS–AA^5^ parameters with 1.14*CMIA partial atomic charges were generated by LigParGen^6-8^ for the organic solvent molecules.^9^ The charges and force fields for TFSI anions in the electrolytes were taken from previous publications.^10^ The OPLSAA force field developed by Jorgensen was used for Ca^2+^ ions. To get the electrolyte structure, isothermal-isobaric (NPT) ensemble runs were first performed at 330 K for 1.5 ns and then 298 K for 1 ns to ensure the equilibrium salt dissociation. Then, 6.5 ns canonical (NVT) ensemble simulations were performed, with the system temperature maintained at 298 K. Both the NPT and NVT ensembles were regulated using the Nosé–Hoover method. Long-range electrostatic interactions were treated with the particle–particle particle–mesh (PPPM) approach, and Lorentz–Berthelot mixing rules were applied to describe cross-interactions between different atom types. The time step for all simulations was set to 1 fs. The last 0.5 ns were analyzed using Python codes to count the number of anions and solvent molecules surrounding each Ca^2+^ and to calculate the percentages of all solvation structures.

The radial distribution function (RDF, g(r)) and coordination number (CN) were calculated from the molecular dynamics trajectories obtained with LAMMPS. The RDF was computed between Ca^2+^ and the oxygen atoms in DME solvent molecules and TFSI^−^ anions, with a cutoff distance of 12 Å. The coordination number was obtained by integrating the RDF up to the first minimum rmin of g(r), according to:

$$CN=4\pi\rho\int_{0}^{r_{\min}} g(r)r^{2}\mathrm{dr}$$

where ρ is the number density of the coordinating species. The integration was performed by post-processing the RDF data using in-house scripts.

Table S1. NBO analysis of compounds and solvation sheath.

|  | Atomic Charge O | Atomic Charge Ca |
| --- | --- | --- |
| Ca^2+^ | - | 2 |
| DME | -0.61509 | - |
| [Ca(DME)₄]²⁺ | -0.68857 | 1.86021 |

Table S2. The chemical shift in the electrolyte after the addition of ionic liquids as shown by ^1^H NMR

| Number | Sample | CH_2_ (DME) /ppm | CH_3_ (DME) /ppm | CH (EMIM^+^) /ppm | CH_3_ (EMIM^+^)  /ppm |
| --- | --- | --- | --- | --- | --- |
| 1 | DME | 3.56 | 3.40 | - | - |
| 2 | Ca(TFSI)_2_ in DME | 3.54 | 3.37 | - | - |
| 3 | DME+EMIMTFSI | 3.19 | 2.99 | 8.39 | 3.62 |
| 4 | Ca(TFSI)_2_ in DME+EMIIMTFSI | 3.19 | 2.98 | 8.36 | 3.59 |
| 5 | EMIMTFSI | - | - | 7.93 | 3.29 |
| 6 | Ca(TFSI)_2_ in EMIMTFSI | - | - | 7.92 | 3.28 |

Table S3. EXAFS fitting parameters at the Ca K-edge various samples (S_0_^2^=0.76)

| samples | path | C. N.^[a]^ | R (Å) ^[b]^ | σ^2^ (´ 10^−3^ Å^2^) ^[c]^ | ΔE (eV) ^[d]^ | R factor^[e]^ |
| --- | --- | --- | --- | --- | --- | --- |
| CaO | Ca-O | 6 | 2.37 | 8.7 | 0.6 | 0.020 |
| Ca 1 | Ca-O | 4.6 | 2.31 | 5.7 | -3.2 | 0.009 |
| Ca 2 | Ca-O | 4.9 | 2.34 | 6.2 | -2.8 | 0.008 |

*^a^*C. N.: coordination numbers; *^b^R*: bond distance; *^c^σ*^2^: Debye-Waller factors; *^d^*Δ*E*_0_: the inner potential correction.

*^e^R* factor: goodness of fit.

Table S4. The fitted system resistance (Rs) and interfacial resistance R_int_ of Ca before and after cycling in CTFSI-DME and CTFSI-DME/EMIMTFSI.

|  | R_s_(Ω) | Interfacial resistance R_int_ (Ω) |
| --- | --- | --- |
|  |  | R_ct_ |
| 0.1M Ca(TFSI)_2_ in DME | 108.5 | 1.75e6 |
| 0.1M Ca(TFSI)_2_ in DME/EMIMTFSI | 108.8 | 133630 |

Table S5. The fitted system resistance (Rs) and interfacial resistance R_int_ of Ca before and after cycling in CTFSI-DME/EMIMOTf.

|  | R_s_(Ω) | Interfacial resistance R_int_ (Ω) | |
| --- | --- | --- | --- |
|  |  | R_SEI_ | R_ct_ |
| 0.1M Ca(TFSI)_2_ in DME/EMIMOTf | 2.915 | - | 63531 |
|  | 4.363 | 42.57 | 26094 |

Table S6. Summary of electrolyte formulations and some electrochemical performance and parameters in previous calcium metal battery reports at room temperature.

| Electrolytic properties | Electrodes | Anodic stability/V | Plating/stripping over-potential/V | Reference |
| --- | --- | --- | --- | --- |
| 0.1 M Ca(TFSI)_2_ in 50:50 vol% DME:EMIMOTf | Ca\|\|Ca | 4.45 (Al) | ~0.26  (0.02 mA cm^-2^) | our work |
| 1.5 M Ca(BH_4_)_2_ | WE: Au, CE and RE: Ca. | ~3.0 (Au) | -0.25/0.10  (25 mV s^-1^) | *Nat. Mater.* 2018, 17,16−20. |
| 0.25 M Ca[B(hfip)_4_]_2_ in DME | WE: Pt, CE and RE: Ca. | 3.9 (Pt), 4.5 (Al) | -0.22/0.10  (80 mV s^-1^) | *Energy Environ. Sci.*, 2019, 12, 3496. |
| [Ca(BH_4_)_2_]_0.05_[N_07_TFSI]_0.95_ ionic liquid | WE: Au, CE and RE: Ca. | - | -1.25  (20mV s^-1^) | *Energy Environ. Sci.*, 2020, 13, 2559. |
| 1 M Ca(BF_4_)_2_ in EMIMOTf | WE: Cu,  CE: Pt, RE: Ca. | 3 (Cu) | -0.34/1.52  (25 mV s^-1^) | *ACS Appl. Energy Mater.* 2020, 3, 3, 2310–2314 |
| 0.5 M CMC/DME/THF | WE: Au, CE and RE: Ca. | ~4.5 (Au) | -0.22/0.08  (20 mV s^-1^) | *Scientific Reports*, 2021, 11, 7563. |
| 0.1 M Ca(TFSI)_2_ in DMAc | Ca\|\|Ca | - | ~0.50  (0.02 mA cm^-2^) | *Angew. Chem. Int. Ed.* 2022, e202214796. |
| Ca(CB_11_H_12_)_2_ in DME/THF | WE: Au, CE and RE: Ca. | - | -0.2/0.1  (20 mV s^-1^) | *Adv. Sci.* 2023, 10, 2301178. |
| 0.3 M CaAlhfip in DME | SS\|\|Ca | ~4.1 (SS, ACN as solvent) | ~0.50  (25 mV s^-1^) | *J. Mater. Chem. A*,2023,11, 14738. |
| 0.5 M Ca(TFSI)_2_ in 50:50 vol% DMSO:EMIM-BF_4_ | Ca\|\|Ca | ~4.0 (CNT electrode) | 0.4 (0.2 mA cm^-2^) | *Nature*,2024, 626, 313–318. |
| 0.1 M CaI_2_ in G1/THFB (9 :1 in weight) | Ca\|\|Ca | ~3.1 (SS) | 0.35 (0.05 mA cm^-2^) | *Angew.Chem. Int.Ed*. 2025, 64,e202413416. |

Figure S1. Pie chart of distribution of different solvated structures in 0.1M Ca(TFSI)_2_ in DME (Solvation structures containing anions are defined as CIPs, while those with solvent-exclusive coordination are correspondingly defined as SSIPs).

Figure S2. Molecular models used to calculate binding energy. a) AMIM^+^-DME; b) BMIM^+^-DME; c) C_2_PY^+^-DME; d) EMIM^+^-DME; e) PP13^+^-DME; f) PYr14^+^-DME; g) TEA^+^-DME.

Figure S3. Nyquist EIS plots for SS/SS cells in different electrolytes at room temperature, measured at 10^5^-0.1 Hz with 5 mV amplitude.

Figure S4. Electrostatic potential comparisons of a) EMIM^+^ and b) DME. c) The effect of ion-dipole interaction between DME and EMIM^+^.

Figure S5. a) Single-pulse ^1^H-NMR spectra of CTFSI-DME,CTFSI-EMIMTFSI and CTFSI-DME/EMIMTFSI. b) NOESY experimental full spectrum. c) Enlarge the NOESY spectrum in the range of 6.5-9.0 ppm on the horizontal axis and 1.0-4.5 ppm on the vertical axis d) NOESY experiment full spectrum for reducing signal strength

**a)**

**b)**

**c)**

Figure S6. a) The normalized Ca K-edge XANES spectra of CaO, CTFSI-DME and CTFSI-DME/EMIMTFSI; Inset: the enlarged Ca K-edge XANES spectra. b) The corresponding EXAFS spectra in R space and c) wavelet transform images of the EXAFS spectra.

Figure S7. Pie chart of distribution of different solvated structures in CTFSI-DME/EMIMTFSI (Solvation structures containing anions are defined as CIPs, while those with solvent-exclusive coordination are correspondingly defined as SSIPs).

Figure S8. Changes of the Ca^2+^ solvation structure from SSIP to CIP-rich electrolyte.

Figure S9. Bond length changes of DME in different solvated structures.

Figure S10. RDF profiles demonstrating the coordination microstructures of a) O(DME)-N(EMIM^+^) in 0.1M Ca(TFSI)_2_ in DME/EMIMTFSI; O(DME)-Ca in b) 0.1M Ca(TFSI)_2_ in DME and c) 0.1M Ca(TFSI)_2_ in DME/EMIMTFSI.

Figure S11. The radical distribution function g(r) analyses of a) CTFSI-DME, b) CTFSI-DME/EMIMTFSI and c) CTFSI-DME/EMIMOTf. Pie chart of distribution of different solvated structures in d) CTFSI-DME, e) CTFSI-DME/EMIMTFSI and f) CTFSI-DME/EMIMOTf (Solvation structures containing anions are defined as CIPs, while those with solvent-exclusive coordination are correspondingly defined as SSIPs).

Figure S12. Negative-going linear sweep voltammogram (scan rate: 1 mV/s).

Figure S13. Electrolyte exchange experiments to discern the role of SEI in the electrolyte compatibility with Ca metal anodes.

We first disassembled cells cycled in a compatible electrolyte (e.g., CTFSI-DME/EMIMTFSI) to harvest SEI-coated calcium anodes (denoted Ca@SEI). These Ca@SEI electrodes were then reassembled into new cells containing an incompatible electrolyte (CTFSI/DME) for testing. The Ca@SEI electrodes exhibited poor cycling stability with significant voltage fluctuations in CTFSI/DME electrolytes. This degradation is attributed to the solvent coordination structure of CTFSI/DME, where each Ca^2+^ ion is solvated by four DME molecules. The SEI formed in modified electrolytes proved insufficient to suppress continuous decomposition of excess DME at the interface. Despite targeting both solvation structure and interface protection, the persistently high coordination of DME molecules in CTFSI/DME electrolyte remains unchanged, constituting the fundamental limitation. These results demonstrate that the EMIMTFSI-induced Ca2+ solvation restructuring is critical, with modified desolvation behavior playing a more dominant role than interfacial properties in governing overpotential and cycling stability.

Figure S14. 3D reconstruction distribution mappings on the Ca metal anode surface after cycling in the three electrolytes measured by TOF-SIMS.

Figure S15. COMSOL simulation results of a) CTFSI-DME and b) CTFSI-DME/EMIMTFSI.

Figure S16. Equivalent circuit model R(RQ) for fitting Nyquist plots of CTFSI-DME/EMIMTFSI. R_s_ is the system resistance. CPE is the constant phase element. R_ct_ is the resistance corresponding to the charge transfer.

Figure S17. LSV of a) Ca//SS, b) Ca//Ti, c) Ca//Al asymmetric cells in CTFSI-DME/EMIMTFSI electrolyte. d) Oxidation potential with error bars of Ca//SS (Al or Ti) asymmetric cells in CTFSI-DME/EMIMTFSI electrolyte.

Figure S18. Galvanostatic cycling stability of Ca//Ca symmetric cell in CTFSI-DME/EMIMTFSI electrolyte at a current density of 0.04 mA cm⁻^2^ with a capacity density of 0.04 mA h cm⁻^2^.

Figure S19. The radical distribution function g(r) analysis of CTFSI-DME/EMIMOTf.

Figure S20. Pie chart of distribution of different solvated structures in CTFSI-DME/EMIMOTf (Solvation structures containing anions are defined as CIPs, while those with solvent-exclusive coordination are correspondingly defined as SSIPs).

Figure S21. SEM image of bare Ca anode after 10 cycles in CTFSI-DME/EMIMOTf.

4.217	4.446

Figure S22. LSV of Ca//SS or Al asymmetric cells in CTFSI-DME/EMIMOTf electrolyte.

Figure S23. Equivalent circuit model R(RQ)(RQ) for fitting Nyquist plots of CTFSI-DME/EMIMOTf. R_s_ is the system resistance. CPE is the constant phase element. R_SEI_ is the resistance corresponding to the ion migration in SEI. R_ct_ is the resistance corresponding to the charge transfer.

Figure S24. Tafel plots in CTFSI-DME and CTFSI-DME/EMIMOTf.

Figure S25. Galvanostatic cycling stability of Ca//Ca symmetric cell in CTFSI-DME/EMIMOTf electrolyte at a current density of 0.04 mA cm⁻^2^ with a capacity density of 0.04 mA h cm⁻^2^.

Figure S26. Charge/discharge curves in PTCDI//Ca full cell at a current density of 10 mA g^-1^ in CTFSI-DME/EMIMTFSI electrolyte within the voltage range of 0.4-3.0 V.

**Supporting References**

1. M. J. Frisch, G. W. Trucks, H. B. Schlegel, G. E. Scuseria, M. A. Robb, J. R. Cheeseman, G. Scalmani, V. Barone, G. A. Petersson, H. Nakatsuji, X. Li, M. Caricato, A. V. Marenich, J. Bloino, B. G. Janesko, R. Gomperts, B. Mennucci, H. P. Hratchian, J. V. Ortiz, A. F. Izmaylov, J. L. Sonnenberg, Williams, F. Ding, F. Lipparini, F. Egidi, J. Goings, B. Peng, A. Petrone, T. Henderson, D. Ranasinghe, V. G. Zakrzewski, J. Gao, N. Rega, G. Zheng, W. Liang, M. Hada, M. Ehara, K. Toyota, R. Fukuda, J. Hasegawa, M. Ishida, T. Nakajima, Y. Honda, O. Kitao, H. Nakai, T. Vreven, K. Throssell, J. A. Montgomery Jr., J. E. Peralta, F. Ogliaro, M. J. Bearpark, J. J. Heyd, E. N. Brothers, K. N. Kudin, V. N. Staroverov, T. A. Keith, R. Kobayashi, J. Normand, K. Raghavachari, A. P. Rendell, J. C. Burant, S. S. Iyengar, J. Tomasi, M. Cossi, J. M. Millam, M. Klene, C. Adamo, R. Cammi, J. W. Ochterski, R. L. Martin, K. Morokuma, O. Farkas, J. B. Foresman and D. J. Fox, Journal, 2016.
2. A. V. Marenich, C. J. Cramer and D. G. Truhlar, The Journal of Physical Chemistry B, 2009, 113, 6378-6396.
3. A. E. Reed, L. A. Curtiss and F. Weinhold, Chemical Reviews, 1988, 88, 899-926.
4. T. Lu and F. Chen, J Comput Chem, 2012, 33, 580-592.
5. W. L. Jorgensen, D. S. Maxwell and J. Tirado-Rives, Journal of the American Chemical Society, 1996, 118, 11225-11236.
6. W. L. Jorgensen and J. Tirado-Rives, Proc Natl Acad Sci U S A, 2005, 102, 6665-6670.
7. L. S. Dodda, J. Z. Vilseck, J. Tirado-Rives and W. L. Jorgensen, J Phys Chem B, 2017, 121, 3864-3870.
8. J. Wang, W. Wang, P. A. Kollman and D. A. Case, J Mol Graph Model, 2006, 25, 247-260.
9. M. S. Kelkar and E. J. Maginn, J Phys Chem B, 2007, 111, 4867-4876.
10. D. Beglov and B. Roux, The Journal of Chemical Physics, 1994, 100, 9050-9063.
